# Our Contemporaries

**Published:** 1917-02

**Authors:** 


### OUR CONTEMPORARIES

---

The Canadian Practitioner & Review reprints in its Jan. issue, our editorial on "Doing Nicely;" the Memphis Medical Journal and the N. Y. Medical Journal, that entitled "Those

Were Happy Days." The latter editorial narrowly missed the waste basket and we are naturally gratified that more impartial criticism was less strict than our own.

---

The Archives Medicales Belges, is issued from the Cabour Military Hospital, Adinkerke, beginning with Jan. 1917.

---

Beginning with the January issue, the Providence Medical Journal, supported by the Providence Medical Assn., becomes the Rhode Island Medical Journal, conforming in size of page and advertising standards to the state journals generally. Dr. Roland Hammond and Dr. J. F. Hawkins of Providence are, respectively, editor and business manager.

---

Dr. Philip Schrainka, of St. Louis, has severed his connection with the Interstate Medical Journal and will start a journal of his own, known as Medicine and Surgery, the first issue being in February 1917.

